# Psychological distress and voting behaviour in nine countries of the former Soviet Union

**DOI:** 10.1038/s41598-023-49071-8

**Published:** 2023-12-19

**Authors:** Andrew Stickley, Tomiki Sumiyoshi, Naoki Kondo, Mall Leinsalu, Yosuke Inoue, Vladislav Ruchkin, Jae Il Shin, Martin McKee

**Affiliations:** 1 Stockholm Centre for Health and Social Change (SCOHOST), Sodertorn University, 141 89 Huddinge, Sweden; 2 Department of Preventive Intervention for Psychiatric Disorders, National Center of Neurology and Psychiatry, Kodaira, Tokyo Japan; 3 Department of Social Epidemiology, Graduate School of Medicine and School of Public Health, Kyoto University, Kyoto, Japan; 4 Department of Epidemiology and Biostatistics, National Institute for Health Development, Tallinn, Estonia; 5 Department of Epidemiology and Prevention, Center for Clinical Sciences, National Center for Global Health and Medicine, Tokyo, Japan; 6 Department of Medical Sciences, Unit of Child and Adolescent Psychiatry, Uppsala University, Uppsala, Sweden; 7 Sala Forensic Psychiatric Clinic, Sala, Sweden; 8 Department of Pediatrics, Yonsei University College of Medicine, Yonsei-ro 50, Seodaemun-gu, Seoul, South Korea; 9 Department of Health Services Research and Policy, London School of Hygiene & Tropical Medicine, London, UK

**Keywords:** Human behaviour, Psychiatric disorders

## Abstract

Poorer mental health is linked to a lower likelihood of voting in elections. However, little is known about this association in non-Western settings. This study examined the association between psychological distress and voting in nine countries of the former Soviet Union (FSU). Data were analysed from 18,000 respondents aged ≥ 18 in Armenia, Azerbaijan, Belarus, Georgia, Moldova, Kazakhstan, Kyrgyzstan, Russia and Ukraine collected during the Health in Times of Transition (HITT) survey in 2010/11. Information was collected on previous voting behaviour and future voting intentions. Psychological distress was assessed with a 12-item scale. In pooled multivariable logistic regression analyses psychological distress was significantly associated with ‘never voting’ (not having voted previously or intending to vote in future) and ‘past voting only’ (having voted previously but not intending to vote in future). In stratified analyses psychological distress was linked to never voting in women and working-age adults. The significant association between psychological distress and voting was observed only in hybrid political regimes. Psychological distress is associated with a reduced likelihood of voting in FSU countries especially among women, working-age adults and those in hybrid political regimes.

## Introduction

In 2019 an estimated 970 million people were living with a mental disorder worldwide^1^. Common mental disorders such as anxiety and depression are especially prevalent^1^ with a recent study of 30 countries reporting an aggregate point prevalence of depression of 12.9%^2^. Although estimates from the Global Burden of Disease study point to stability in the age-standardised incidence rate of depression worldwide between 1990 and 2017, the number of incident cases nevertheless increased by 49.9%^3^. This is worrying given that poor mental health impacts negatively on functioning across the life course. While many of the relationships are bidirectional, it has long been recognised that worse mental health is linked, for example, to poorer adjustment to school and worse academic performance^4^, reduced employment in adulthood^5^ and an increased risk of marital dissolution^6^. Individuals with mental disorders are also more likely to have co-occurring physical health conditions^7^ and die prematurely^8,9^.

One consequence of poor mental health that has, until recently, received less attention is its role in the democratic process. While more countries are holding elections, voter turnout has been declining since the 1990s^10^, causing concern about the health of some democracies^11^. Most research on voter turnout has focused on individuals’ ability and motivation to vote and the barriers to doing so^12^. However, some recent work has also found a link between mental health and voting behaviour. One study using data from four cross-sectional and longitudinal surveys from 25 European countries, Israel and the United States found that depressive symptoms were linked to reduced voting in three of the four datasets and were of near significance in the fourth (p < 0.10)^13^. An earlier study using individual-level register data linked depression and psychotic mental disease with a lower probability of voting in the 1999 Finnish parliamentary election^14^. Other research from the United States has shown that depressed mood/depressive symptoms in adolescence (for subsequent turnout), adulthood^15^ and among older adults^16^ are also linked to not voting and that the inverse association between depressive symptoms and voting does not differ by gender or race^17^. Other studies find a stronger effect of poor mental health on voting in local than in national elections^18^, that depression reduces the probability of voting over time^19^, and that reduced political efficacy may mediate the association between depression and voting behaviour^20^. It is important to note however, that not all studies have found an association between worse mental health and voting behaviour in all instances. Denny and Doyle found a significant relationship between poor mental health and reduced voting in only one of three British general elections^21^, while an ecological study found no association between poorer mental health and county-level voting in Georgia, United States, across two election cycles and that it was *positively* associated with voting in a third^22^.

In this study we will examine the association between mental health and voting behaviour in nine countries of the former Soviet Union (FSU). Most research to date has focused on Western democracies, with comparatively little attention given to countries that are not fully democratic or non-democratic. Indeed, in the year our data were collected (2010) none of our study countries were ranked as full democracies in the Democracy Index but were rather ‘flawed democracies’ (Moldova, Ukraine), ‘hybrid regimes’ (Kyrgyzstan, Georgia, Russia, Armenia) or ‘authoritarian regimes’ (Belarus, Kazakhstan, Azerbaijan), with political participation index scores (that included voter participation) ranging from 6.11/10.00 (Moldova) to 3.33 (Belarus, Kazakhstan, Azerbaijan)^23^ (see online Appendix 1). It is possible that the association between mental health and the propensity to vote may differ in these countries given the differences from Western democracies. For example, levels of trust in state institutions are low^24,25^ and clientelism i.e. the practice of exchanging goods and services for votes, is reportedly common in this region^26^. Additionally, coercion may influence voter turnout in authoritarian regimes^27^—which might explain why reported turnout was over 90% in the 2006 and 2010 Belarus presidential elections^28^, and it is possible that in such circumstances people with mental health problems might be forced to vote. Indeed, it has been argued that elections in such regimes are mere ‘window dressing’ and devoid of meaningful participation^27^. However, other research has indicated that voter turnout can vary in authoritarian regimes. Thus, compared to in Belarus, a much smaller share of voters (71.6%) participated in the 2013 Azerbaijan presidential election^29^. Moreover, a recent study using data from all our study countries except Moldova, collected between 2017 and 2020, reported that ‘never’ voting in national elections was more prevalent in authoritarian than hybrid regimes (16.4% > 12.1%)^30^. Research from Russia also indicates that many reasons for not voting in national elections, such as a lack of political efficacy, trust and interest in politics^31^ mirror those in European democracies^32^. In short, voting behaviour in our study countries is affected by a range of factors that are sometimes, but not always, the same as in Western democracies. As yet, there has been little indication of whether and how this might affect the mental health-voting association although a recent study indicated that the effects of depression on the voting gap might differ between FSU countries such as Russia and Ukraine and many other European countries^13^.

Thus, the main aim of this study is to examine the association between psychological distress and voting behaviour in nine FSU countries. A focus on psychological distress is warranted given that most research to date has examined depression and voting behaviour, even though a need to consider other mental health problems has been noted^19^. Psychological distress is common in the general population and has been defined as “a state of emotional suffering characterised by symptoms of depression…and anxiety”^33^ and linked to a variety of negative outcomes including multimorbidity^34^ and premature mortality^35^. A second aim of this study is to determine if there are sex and/or age differences in any associations. This is necessary as there is evidence that there may be age and sex differences in voting in some elections^36,37^ including in the FSU countries^38,39^. Moreover, an earlier study also found age and sex differences in psychological distress in the FSU countries^40^. It is possible that common mechanisms may underlie differences in voting behaviour and poorer mental health. For instance, the gender wage gap that exists in the FSU countries^41^ might be important given that in a recent Italian study, women were more likely to abstain from voting than men and labour market (wage) inequalities were linked to non-voting^42^, while earlier research also found that women receiving lower wages than their male counterparts were more likely to have major depressive disorder and generalised anxiety disorder^43^. Finally, we will also explore whether the association between mental health and voting behaviour differs by the type of political regime and in the individual countries.

As the mental health-participation gap may reduce policy representation and affect mental illness stigma^44^, furthering understanding of the mental health-voting behaviour association may have important public health implications.

## Results

### Descriptive statistics

Just over three-quarters of the respondents reported having voted previously and intending to do so in future (i.e. they were always voters, 76.1%, N = 13,669). Many fewer reported that they had not voted previously and would not vote in the future (i.e. never voters, 11.1%, N = 1992). Just over 5% of the respondents reported not voting previously but that they would do so in the future (i.e. future voters only, 5.2%, N = 928), while 7.6% (N = 1372) reported that they had previously voted but would not do so in future (i.e. past voters only). The sample characteristics stratified by voting behaviour status are presented in Table 1.

**Table 1:** Basic characteristics of the study sample after multiple imputation.

Variable	Total	Voted and will vote in future	Voted but will not vote in the future	Did not vote but will in the future	Did not vote, will not in future
	(N = 18,000)	(N = 13,669^a^)	(N = 1372^b^)	(N = 928^c^)	(N = 1992^d^)
Sex					
Men	43.5	43.3	44.0	50.4	41.5
Women	56.5	56.7	56.0	49.6	58.5
Age					
18–34	37.8	35.0	34.5	70.4	43.7
35–59	43.3	44.8	44.0	24.2	41.2
≥ 60	18.9	20.2	21.5	5.3	15.1
Education					
High	27.5	28.3	26.9	25.3	23.8
Mid	59.4	58.6	60.2	62.3	63.0
Low	13.1	13.1	13.0	12.4	13.2
Marital status					
Married/cohabiting	62.0	64.4	59.2	43.2	56.5
Never married	20.6	18.0	19.6	50.1	25.1
Divorced/widowed	17.4	17.6	21.2	6.7	18.4
Household finances					
Good/very good	22.4	22.0	21.9	31.7	21.3
Average	57.3	58.2	52.5	54.5	55.8
Bad/very bad	20.3	19.8	25.6	13.8	23.0
Live alone					
No	90.6	90.6	87.8	95.9	90.0
Yes	9.4	9.4	12.2	4.1	10.0
Location					
Urban	60.4	58.3	66.5	67.1	67.4
Rural	39.6	41.7	33.5	32.9	32.6
Self-rated health					
Very good/good	40.6	38.7	39.3	63.5	43.8
Fair	40.9	42.3	40.5	28.9	37.7
Poor/very poor	18.5	19.0	20.2	7.6	18.4
Low social support					
No	89.0	91.1	77.7	84.7	84.4
Yes	11.0	8.9	22.3	15.3	15.6
Political distrust					
Mean	4.7	4.6	5.0	4.4	4.9
Psychological distress					
No	95.0	95.4	92.9	97.0	93.4
Yes	5.0	4.6	7.1	3.0	6.6

^a^Sample sizes of the imputed datasets varied between 13,669 and 13,703.

^b^Sample sizes of the imputed datasets varied between 1372 and 1387.

^c^Sample sizes of the imputed datasets varied between 928 and 944.

^d^Sample sizes of the imputed datasets varied between 1992 and 2012.

### Psychological distress and never voting

In the bivariate analysis, psychological distress was associated with 75% higher odds for never voting in the total sample (OR: 1.75, 95%CI: 1.43–2.15) (Model 1, Table 2). Including sociodemographic variables and self-rated health in the analysis had a small effect, while the inclusion of low social support in Model 4 and political distrust in Model 5 further reduced the odds. In the fully adjusted analysis psychological distress continued to be significantly associated with never voting (OR: 1.52, 95%CI: 1.21–1.89). Other variables associated with this outcome included younger age, having a mid or a low education, being never married or divorced/widowed, urban residency, low social support and greater political distrust.

**Table 2:** Association between psychological distress and never voting (not having voted in the past and planning not to vote in the future) in nine countries of the former Soviet Union (N = 15,672^a^).

	Model 1	Model 2	Model 3	Model 4	Model 5
	OR (95%CI)	OR (95%CI)	OR (95%CI)	OR (95%CI)	OR (95%CI)
Psychological distress	1.75 (1.43–2.15)***	1.70 (1.38–2.10)***	1.68 (1.35–2.08)***	1.61 (1.29–2.01)***	1.52 (1.21–1.89)***
Sex (woman)		1.05 (0.95–1.16)	1.05 (0.95–1.16)	1.06 (0.96–1.18)	1.11 (1.00–1.23)*
Age					
18–34		Ref.	Ref.	Ref.	Ref.
35–59		0.74 (0.65–0.84)***	0.75 (0.66–0.85)***	0.73 (0.65–0.84)***	0.74 (0.65–0.84)***
≥ 60		0.50 (0.41–0.59)***	0.50 (0.41–0.60)***	0.49 (0.40–0.59)***	0.54 (0.44–0.65)***
Education					
High		Ref.	Ref.	Ref.	Ref.
Mid		1.31 (1.16–1.48)***	1.31 (1.16–1.48)***	1.29 (1.14–1.45)***	1.35 (1.19–1.52)***
Low		1.64 (1.36–1.97)***	1.63 (1.35–1.95)***	1.56 (1.30–1.88)***	1.73 (1.44–2.09)***
Marital status					
Married/cohabiting		Ref.	Ref.	Ref.	Ref.
Never married		1.25 (1.09–1.44)**	1.25 (1.08–1.43)**	1.23 (1.07–1.41)**	1.23 (1.07–1.42)**
Divorced/widowed		1.26 (1.07–1.47)**	1.26 (1.07–1.47)**	1.19 (1.02–1.40)*	1.20 (1.02–1.40)*
Household finances					
Good/very good		Ref.	Ref.	Ref.	Ref.
Average		1.03 (0.91–1.18)	1.04 (0.92–1.19)	1.03 (0.91–1.18)	0.96 (0.84–1.10)
Bad/very bad		1.39 (1.17–1.64)***	1.39 (1.17–1.65)***	1.29 (1.09–1.53)**	1.08 (0.90–1.28)
Live alone		1.12 (0.92–1.36)	1.11 (0.91–1.35)	1.04 (0.85–1.26)	1.06 (0.86–1.29)
Location (rural)		0.72 (0.64–0.80)***	0.72 (0.64–0.80)***	0.71 (0.63–0.79)***	0.74 (0.67–0.83)***
Self-rated health					
Good/very good			Ref.	Ref.	Ref.
Fair			0.93 (0.82–1.05)	0.93 (0.83–1.05)	0.90 (0.79–1.01)
Poor/very poor			1.01 (0.86–1.20)	1.01 (0.85–1.20)	0.98 (0.82–1.16)
Low social support				1.92 (1.65–2.24)***	1.84 (1.57–2.14)***
Political distrust					1.17 (1.14–1.20)***

OR: odds ratio; CI: confidence interval; Ref: reference category.

***p < 0.001, **p < 0.01, *p < 0.05 (Wald test).

All models were adjusted for country.

^a^The sample size of the imputed datasets varied between 15,672 and 15,696.

### Psychological distress and past voting only

Psychological distress was associated with 65% higher odds for past voting only in the bivariate analysis (OR: 1.65, 95%CI: 1.31–2.09) (Model 1, Table 3).This association was attenuated after adjusting for the sociodemographic variables, self-rated health and low social support (Models 2–4) but remained significant (OR: 1.36. 95%CI: 1.06–1.75). Adjustment for political distrust (Model 5) further attenuated this association although it remained statistically significant in the fully adjusted analysis (OR: 1.29, 95%CI: 1.00–1.67). Very few variables were associated with past voting only in Model 5. Specifically, having average household finances was associated with lower odds for past voting only (OR: 0.85, 95%CI: 0.73–0.99), while both low social support (OR: 2.53, 95%CI: 2.16–2.96) and greater political distrust (OR: 1.12, 95%CI: 1.09–1.16) were associated with significantly higher odds for past voting only.

**Table 3:** Association between psychological distress and past voting only (having voted in the past but planning not to vote in the future) in nine countries of the former Soviet Union (N = 15,050^a^).

	Model 1	Model 2	Model 3	Model 4	Model 5
	OR (95%CI)	OR (95%CI)	OR (95%CI)	OR (95%CI)	OR (95%CI)
Psychological distress	1.65 (1.31–2.09)***	1.46 (1.15–1.86)**	1.46 (1.14–1.87)**	1.36 (1.06–1.75)*	1.29 (1.00–1.67)*
Sex (woman)		0.92 (0.81–1.03)	0.92 (0.82–1.03)	0.94 (0.83–1.05)	0.96 (0.86–1.09)
Age					
18–34		Ref.	Ref.	Ref.	Ref.
35–59		0.99 (0.85–1.15)	1.00 (0.86–1.17)	0.97 (0.83–1.14)	0.98 (0.84–1.14)
≥ 60		0.90 (0.74–1.10)	0.91 (0.74–1.13)	0.88 (0.71–1.08)	0.93 (0.76–1.15)
Education					
High		Ref.	Ref.	Ref.	Ref.
Mid		1.01 (0.88–1.15)	1.01 (0.88–1.16)	0.98 (0.85–1.12)	1.02 (0.89–1.17)
Low		1.04 (0.84–1.28)	1.04 (0.84–1.28)	0.98 (0.79–1.21)	1.06 (0.85–1.31)
Marital status					
Married/cohabiting		Ref.	Ref.	Ref.	Ref.
Never married		1.14 (0.95–1.35)	1.13 (0.95–1.35)	1.11 (0.93–1.32)	1.11 (0.93–1.32)
Divorced/widowed		1.18 (0.99–1.41)	1.18 (0.99–1.41)	1.11 (0.92–1.33)	1.11 (0.93–1.33)
Household finances					
Good/very good		Ref.	Ref.	Ref.	Ref.
Average		0.90 (0.78–1.05)	0.91 (0.79–1.06)	0.91 (0.78–1.06)	0.85 (0.73–0.99)*
Bad/very bad		1.40 (1.16–1.68)***	1.41 (1.17–1.71)***	1.27 (1.05–1.54)*	1.10 (0.90–1.34)
Live alone		1.22 (0.98–1.51)	1.21 (0.98–1.51)	1.09 (0.87–1.36)	1.11 (0.89–1.38)
Location (rural)		0.87 (0.76–0.98)*	0.87 (0.76–0.98)*	0.86 (0.76–0.98)*	0.89 (0.79–1.01)
Self-rated health					
Good/very good			Ref.	Ref.	Ref.
Fair			0.94 (0.82–1.08)	0.96 (0.83–1.11)	0.94 (0.81–1.08)
Poor/very poor			0.97 (0.80–1.19)	0.98 (0.80–1.20)	0.96 (0.79–1.18)
Low social support				2.55 (2.18–2.99)***	2.53 (2.16–2.96)***
Political distrust					1.12 (1.09–1.16)***

OR: odds ratio; CI: confidence interval; Ref: reference category.

***p < 0.001, **p < 0.01, *p < 0.05 (Wald test).

All models were adjusted for country.

^a^The sample size of the imputed datasets varied between 15,050 and 15,079.

### Sex- and age-stratified analyses

In a sex-stratified analysis psychological distress was not associated with never voting among men in any of the analyses (Table 4). In contrast, in the bivariate analysis psychological distress was associated with almost two times higher odds for never voting among women (OR: 1.99, 95%CI: 1.49–2.67). Further adjustment for the covariates only slightly attenuated the strength of the association so that in the fully adjusted Model 5 psychological distress was associated with 76% higher odds for never voting among women (OR: 1.76, 95%CI: 1.28–2.41). In the age-stratified analysis psychological distress was associated with higher odds for never voting in all of the age groups in the bivariate analysis with ORs ranging from 1.85 (age 18–34) to 2.23 (age ≥ 60). Further adjustment for the sociodemographic variables, self-rated health, low social support and political distrust attenuated the ORs in all age groups. However, while psychological distress continued to be significantly associated with never voting in those aged 18–34 (OR: 1.51, 95%CI: 1.03–2.22) and adults aged 35–59 (OR: 1.55, 95%CI: 1.11–2.15) in the fully adjusted Model 5, the association had become non-significant in those aged 60 and above (OR: 1.61, 95%CI: 0.92–2.81).

**Table 4:** Sex- and age-specific associations between psychological distress and never voting (not having voted in the past and planning not to vote in the future) in nine countries of the former Soviet Union.

	Model 1	Model 2	Model 3	Model 4	Model 5
	OR (95%CI)	OR (95%CI)	OR (95%CI)	OR (95%CI)	OR (95%CI)
Sex					
Men (N = 6744^a^)					
Psychological distress	1.22 (0.86–1.72)	1.23 (0.86–1.76)	1.14 (0.79–1.65)	1.06 (0.73–1.53)	1.01 (0.69–1.47)
Women (N = 8924^b^)					
Psychological distress	1.99 (1.49–2.67)***	1.92 (1.42–2.60)***	1.92 (1.41–2.62)***	1.86 (1.36–2.54)***	1.76 (1.28–2.41)***
Age					
18–34 (N = 5660^c^)					
Psychological distress	1.85 (1.29–2.65)**	1.69 (1.17–2.45)**	1.70 (1.16–2.49)**	1.63 (1.11–2.40)*	1.51 (1.03–2.22)*
35–59 (N = 6951^d^)					
Psychological distress	1.92 (1.41–2.60)***	1.75 (1.28–2.40)***	1.71 (1.23–2.36)**	1.67 (1.20–2.31)**	1.55 (1.11–2.15)*
≥ 60 (N = 3059^e^)					
Psychological distress	2.23 (1.31–3.80)**	1.80 (1.05–3.09)*	1.74 (1.00–3.00)*	1.64 (0.94–2.84)	1.61 (0.92–2.81)

Model 1 examined the bivariate association between psychological distress and never voting; Model 2 additionally adjusted for sex and age (where

appropriate), education, marital status, household finances, living alone, location; Model 3 was additionally adjusted for self-rated health; Model 4

was additionally adjusted for low social support; Model 5 was additionally adjusted for political distrust.

All models were adjusted for country.

OR: odds ratio; CI: confidence interval.

***p < 0.001, **p < 0.01, *p < 0.05 (Wald test).

^a^The sample size of the imputed datasets varied between 6744 and 6756.

^b^The sample size of the imputed datasets varied between 8924 and 8942.

^c^The sample size of the imputed datasets varied between 5660 and 5673.

^d^The sample size of the imputed datasets varied between 6951 and 6963.

^e^The sample size of the imputed datasets varied between 3059 and 3068.

In a bivariate analysis psychological distress was associated with past voting only among women (OR: 1.88, 95%CI: 1.34–2.64) but not men (OR: 1.37, 95%CI: 0.95–1.97) (Model 1, Table 5). However, after adjusting the analysis for low social support (Model 4) the association also became non-significant for women (OR: 1.43, 95%CI: 0.99–2.05). In the age-stratified analysis, psychological distress was associated with past voting only in those aged 35–59 and 60 and above but not in individuals aged 18–34 in Model 1. This association became non-significant in adults aged 35–59 after adjusting for the sociodemographic variables in Model 2 (OR: 1.40, 95%CI: 0.97–2.01) and in those aged 60 and above after adjusting for political distrust in Model 5 (OR: 1.67, 95%CI: 0.97–2.89).

**Table 5:** Sex- and age-specific associations between psychological distress and past voting only (having voted in the past but planning not to vote in the future) in nine countries of the former Soviet Union.

	Model 1	Model 2	Model 3	Model 4	Model 5
	OR (95%CI)	OR (95%CI)	OR (95%CI)	OR (95%CI)	OR (95%CI)
Sex					
Men (N = 6519^a^)					
Psychological distress	1.37 (0.95–1.97)	1.26 (0.87–1.84)	1.32 (0.89–1.94)	1.18 (0.80–1.76)	1.14 (0.76–1.69)
Women (N = 8529^b^)					
Psychological distress	1.88 (1.34–2.64)***	1.54 (1.09–2.19)*	1.51 (1.06–2.15)*	1.43 (0.99–2.05)	1.34 (0.93–1.94)
Age					
18–34 (N = 5257^c^)					
Psychological distress	1.03 (0.59–1.79)	1.02 (0.58–1.77)	1.05 (0.60–1.84)	1.00 (0.56–1.76)	0.92 (0.52–1.64)
35–59 (N = 6729^d^)					
Psychological distress	1.53 (1.07–2.19)*	1.40 (0.97–2.01)	1.37 (0.94–2.00)	1.29 (0.88–1.89)	1.23 (0.84–1.81)
≥ 60 (N = 3051^e^)					
Psychological distress	2.28 (1.36–3.83)**	1.85 (1.09–3.12)*	1.83 (1.08–3.12)*	1.76 (1.02–3.02)*	1.67 (0.97–2.89)

Model 1 examined the bivariate association between psychological distress and past voting only; Model 2 additionally adjusted for sex and age (where

appropriate), education, marital status, household finances, living alone, location; Model 3 was additionally adjusted for self-rated health; Model 4

was additionally adjusted for low social support; Model 5 was additionally adjusted for political distrust.

All models were adjusted for country.

OR: odds ratio; CI: confidence interval.

***p < 0.001, **p < 0.01, *p < 0.05 (Wald test).

^a^The sample size of the imputed datasets varied between 6519 and 6536.

^b^The sample size of the imputed datasets varied between 8529 and 8550.

^c^The sample size of the imputed datasets varied between 5257 and 5278.

^d^The sample size of the imputed datasets varied between 6729 and 6746.

^e^The sample size of the imputed datasets varied between 3051 and 3065.

### Psychological distress and voting in different types of political regime/individual countries

In fully adjusted analyses, psychological distress was not associated with never voting in flawed democracies (OR: 1.59, 95%CI: 0.79–3.19) or authoritarian regimes (OR: 1.00, 95%CI: 0.68–1.47) but was significantly associated with never voting in hybrid regimes (OR: 1.59, 95%CI: 1.16–2.19) (Table 6). At the country level there was only one significant association—in Georgia, where psychological distress was associated with over twice the odds for never voting (OR: 2.24, 95%CI: 1.14–4.39), seemingly underpinning the significant association observed in hybrid regimes.

**Table 6:** Associations between psychological distress and never voting (not having voted in the past and planning not to vote in the future) in different types of political regime and in the individual study countries.

	Model 1	Model 2	Model 3	Model 4	Model 5
	OR (95%CI)	OR (95%CI)	OR (95%CI)	OR (95%CI)	OR (95%CI)
Regime					
Flawed (N = 3405^a^)					
Psychological distress	1.66 (0.89–3.09)	1.56 (0.80–3.06)	1.71 (0.86–3.40)	1.66 (0.83–3.32)	1.59 (0.79–3.19)
Hybrid (N = 7564^b^)					
Psychological distress	1.83 (1.37–2.44)***	1.85 (1.37–2.50)***	1.71 (1.26–2.34)**	1.66 (1.21–2.27)**	1.59 (1.16–2.19)**
Authoritarian (N = 4699^c^)					
Psychological distress	1.36 (0.95–1.94)	1.20 (0.83–1.73)	1.21 (0.83–1.75)	1.12 (0.77–1.63)	1.00 (0.68–1.47)
Country					
Armenia (N = 1349^d^)					
Psychological distress	1.70 (0.78–3.73)	1.62 (0.72–3.62)	1.87 (0.80–4.38)	1.84 (0.78–4.32)	1.86 (0.79–4.38)
Azerbaijan (N = 1574^e^)					
Psychological distress	1.13 (0.67–1.92)	0.95 (0.55–1.66)	1.00 (0.57–1.75)	0.91 (0.51–1.61)	0.83 (0.46–1.50)
Belarus (N = 1603^f^)					
Psychological distress	1.28 (0.65–2.49)	1.23 (0.62–2.44)	1.22 (0.60–2.48)	1.20 (0.59–2.45)	1.08 (0.52–2.22)
Georgia (N = 2001^g^)					
Psychological distress	2.81 (1.53–5.15)**	2.89 (1.51–5.51)**	2.39 (1.24–4.60)**	2.29 (1.19–4.42)*	2.24 (1.14–4.39)*
Kazakhstan (N = 1519^h^)					
Psychological distress	1.34 (0.71–2.55)	1.30 (0.66–2.57)	1.22 (0.61–2.41)	1.16 (0.59–2.31)	1.08 (0.54–2.15)
Kyrgyzstan (N = 1640^i^)					
Psychological distress	1.94 (0.94–4.00)	1.74 (0.81–3.74)	2.04 (0.92–4.52)	1.88 (0.84–4.20)	1.75 (0.78–3.94)
Moldova (N = 1643^j^)					
Psychological distress	1.04 (0.34–3.23)	1.01 (0.31–3.30)	0.96 (0.29–3.21)	0.96 (0.29–3.22)	0.91 (0.27–3.11)
Russia (N = 2567^k^)					
Psychological distress	1.45 (0.93–2.26)	1.52 (0.95–2.42)	1.37 (0.85–2.21)	1.35 (0.83–2.19)	1.36 (0.83–2.24)
Ukraine (N = 1761^l^)					
Psychological distress	1.52 (0.62–3.75)	1.22 (0.46–3.25)	1.44 (0.53–3.92)	1.41 (0.52–3.83)	1.37 (0.50–3.75)

Model 1 examined the bivariate association between psychological distress and never voting; Model 2 was additionally adjusted for sex, age, education, marital status, household finances, living alone, location; Model 3 was additionally adjusted for self-rated health; Model 4 was additionally adjusted for low social support; Model 5 was additionally adjusted for political distrust.

OR: odds ratio; CI: confidence interval.

***p < 0.001, **p < 0.01, *p < 0.05 (Wald test).

^a^The sample size of the imputed datasets varied between 3405 and 3411.

^b^The sample size of the imputed datasets varied between 7564 and 7580.

^c^The sample size of the imputed datasets varied between 4699 and 4713.

^d^The sample size of the imputed datasets varied between 1349 and 1353.

^e^The sample size of the imputed datasets varied between 1574 and 1586.

^f^The sample size of the imputed datasets varied between 1603 and 1607.

^g^The sample size of the imputed datasets varied between 2001 and 2010.

^h^The sample size of the imputed datasets varied between 1519 and 1525.

^i^The sample size of the imputed datasets varied between 1640 and 1641.

^j^The sample size of the imputed datasets varied between 1643 and 1647.

^k^The sample size of the imputed datasets varied between 2567 and 2582.

^l^The sample size of the imputed datasets varied between 1761 and 1765.

A similar result was observed when examining the association between the regime type and past voting only (Table 7). Specifically, while there was no association between psychological distress and past voting only in flawed  democracies (OR: 1.33, 95%CI: 0.72–2.47) or authoritarian regimes (OR: 0.80, 95%CI: 0.46–1.37), psychological distress was significantly associated with past voting only in hybrid regimes (OR: 1.45, 95%CI: 1.03–2.04). When analysed at the country level there was no significant association between psychological distress and past voting only in any country. However, the significant result observed in hybrid regimes may have been due to the association between psychological distress and past voting only in Armenia, where the result was of borderline statistical significance (OR: 2.06, 95%CI: 0.99–4.28).

**Table 7:** Associations between psychological distress and past voting only (having voted in the past but planning not to vote in the future) in different types of political regime and in the individual study countries.

	Model 1	Model 2	Model 3	Model 4	Model 5
	OR (95%CI)	OR (95%CI)	OR (95%CI)	OR (95%CI)	OR (95%CI)
Regime					
Flawed (N = 3444^a^)					
Psychological distress	1.97 (1.15–3.38)*	1.54 (0.86–2.75)	1.67 (0.92–3.03)	1.46 (0.80–2.67)	1.33 (0.72–2.47)
Hybrid (N = 7379^b^)					
Psychological distress	1.75 (1.28–2.40)***	1.65 (1.19–2.28)**	1.57 (1.13–2.20)**	1.50 (1.07–2.11)*	1.45 (1.03–2.04)*
Authoritarian (N = 4221^c^)					
Psychological distress	1.25 (0.75–2.08)	0.95 (0.56–1.62)	0.95 (0.56–1.62)	0.89 (0.52–1.52)	0.80 (0.46–1.37)
Country					
Armenia (N = 1472^d^)					
Psychological distress	1.66 (0.89–3.12)	2.05 (1.05–4.00)*	2.14 (1.06–4.33)*	2.05 (0.99–4.25)	2.06 (0.99–4.28)
Azerbaijan (N = 1228^e^)					
Psychological distress	3.01 (1.42–6.39)**	1.90 (0.82–4.39)	1.65 (0.71–3.86)	1.64 (0.71–3.83)	1.43 (0.60–3.43)
Belarus (N = 1526^f^)					
Psychological distress	0.79 (0.28–2.21)	0.75 (0.26–2.15)	0.79 (0.27–2.31)	0.76 (0.26–2.23)	0.66 (0.22–1.97)
Georgia (N = 1940^g^)					
Psychological distress	2.71 (1.30–5.65)**	2.18 (1.02–4.67)*	2.03 (0.94–4.42)	1.93 (0.88–4.20)	1.91 (0.87–4.21)
Kazakhstan (N = 1458^h^)					
Psychological distress	1.12 (0.50–2.51)	0.78 (0.34–1.82)	0.83 (0.35–1.95)	0.74 (0.31–1.76)	0.72 (0.30–1.73)
Kyrgyzstan (N = 1619^i^)					
Psychological distress	1.28 (0.50–3.27)	1.57 (0.60–4.11)	1.23 (0.46–3.30)	1.12 (0.41–3.03)	1.04 (0.38–2.85)
Moldova (N = 1633^j^)					
Psychological distress	2.64 (1.19–5.82)*	2.00 (0.85–4.68)	2.37 (0.98–5.74)	2.08 (0.84–5.12)	1.91 (0.76–4.78)
Russia (N = 2344^k^)					
Psychological distress	1.64 (0.96–2.81)	1.53 (0.88–2.68)	1.50 (0.85–2.65)	1.48 (0.84–2.62)	1.47 (0.83–2.60)
Ukraine (N = 1809^l^)					
Psychological distress	1.21 (0.50–2.91)	0.87 (0.34–2.23)	0.90 (0.35–2.32)	0.79 (0.30–2.09)	0.72 (0.27–1.92)

Model 1 examined the bivariate association between psychological distress and past voting only; Model 2 was additionally adjusted for sex, age, education, marital status, household finances, living alone, location; Model 3 was additionally adjusted for self-rated health; Model 4 was additionally adjusted for low social support; Model 5 was additionally adjusted for political distrust.

OR: Odds ratio; CI: Confidence interval.

***p < 0.001, **p < 0.01, *p < 0.05 (Wald test).

^a^The sample size of the imputed datasets varied between 3444 and 3449.

^b^The sample size of the imputed datasets varied between 7379 and 7401.

^c^The sample size of the imputed datasets varied between 4221 and 4236.

^d^The sample size of the imputed datasets varied between 1472 and 1475.

^e^The sample size of the imputed datasets varied between 1228 and 1243.

^f^The sample size of the imputed datasets varied between 1526 and 1530.

^g^The sample size of the imputed datasets varied between 1940 and 1950.

^h^The sample size of the imputed datasets varied between 1458 and 1468.

^i^The sample size of the imputed datasets varied between 1619 and 1620.

^j^The sample size of the imputed datasets varied between 1633 and 1636.

^k^The sample size of the imputed datasets varied between 2344 and 2357.

^l^The sample size of the imputed datasets varied between 1809 and 1815.

### Sensitivity analyses

Finally, to determine if our decision to limit the analysis to those individuals in the top 5% of psychological distress scores was important for the observed results, we ran a sensitivity analysis where we instead used the top 10% of scores. Psychological distress continued to be associated with never voting in the total sample (OR: 1.24, 95%CI: 1.03–1.51) but was no longer associated with past voting only (OR: 1.18, 95%CI: 0.96–1.46) (data not shown).

## Discussion

This study examined the association between psychological distress and voting behaviour in nine FSU countries. Over three-quarters of the respondents (76.1%) were categorised as ‘always voters’ who had voted in the past and would do so again in the future. In contrast, 11.1% were ‘never voters’ who had not previously voted and would not do so in the future, while 7.6% reported that they had previously voted but would not do so again in the future ('past voters only'). In fully adjusted pooled multivariable logistic regression analyses psychological distress was associated with significantly higher odds for never voting and past voting only. In stratified analyses psychological distress was linked to never voting in women but not men, and in working-age but not older adults. Psychological distress was associated with both never voting and past voting only in hybrid but not in other types of political regime.

A previous study that used data from over 20 European countries that included Russia and Ukraine found depressive symptoms were linked to lower voter turnout^13^, while other research from Finland^14^ and the United States^15,16^ also found that depression/depressive symptoms were linked to a reduced likelihood of voting. In contrast, a study from the state of Georgia in the United States found that mental illness was not linked to lower voting across three election cycles^22^. Another study that analysed voting behaviour in three general elections in Britain between 1979 and 1997 found that poor mental health, as measured by a ‘malaise inventory score’, was linked to a significantly reduced likelihood of voting in only one of the three elections^21^. The results of the current study provide support for the idea that poorer mental health is associated with a reduced likelihood of voting but also provide some indication that the association is nuanced in that it is not seen in all subpopulations, types of political regime or countries.

It is possible that the elements that are inherent in psychological distress—anxiety and depression^33^—might also play a central role in reduced voting. For example, according to the Diagnostic and Statistical Manual of Mental Disorders—5th Edition, diminished interest in all, or almost all activities, hopelessness, energy loss/fatigue, indecisiveness and a reduced ability to concentrate are all symptoms of major depressive disorder, while fatigue and difficulty concentrating are also symptoms of anxiety disorder^45^. It is feasible that such symptoms may severely inhibit an individual’s desire and/or ability to vote. Indeed, it has been suggested that one way such symptoms might negatively affect voting behaviour is by reducing external political efficacy (i.e. the perceived responsiveness of the political system)^20^ although it should be noted that we found an association between psychological distress and never voting after adjusting for political distrust.

Although previous research has indicated that the association between poorer mental health and voting does not differ by sex^17^, in a sex-stratified analysis we found that psychological distress was associated with never voting among women but not men. As yet, there has been little research on gender differences in the effects of psychological distress although a recent study among older Spanish adults found that psychological distress was associated with worse social functioning in both sexes^46^. Thus, we can only speculate on how distress might be linked to women’s voting behaviour in the FSU countries. For example, this association might emanate from sex-related differences in exposure to stressors and/or the context in which they occur^47^. In particular, women experience a double burden of paid employment and being responsible for all domestic labour and childcare^48^, while retaining primary responsibility for the household garden in informal rural economies^49^. This may be important as there is some evidence that a high unpaid workload in combination with paid employment may result in worse mental health in women—possibly as a result of time poverty^50^, that is, the feeling that there is insufficient free or discretionary time, which has been linked to poorer mental health^51^, and which might also affect a woman’s ability to vote.

In an age-stratified analysis psychological distress was associated with never voting in young and middle-aged but not older adults. Previous studies have been undertaken mostly among adults of all ages^13,18,20^ although some research has focused on the association between mental health and voting in young and middle-aged adults^19,21^, while one study examined the association in working-age adults^14^. The results from these latter studies accord with those from the current study concerning working-age adults. However, another study using data from older adults collected in the Wisconsin Longitudinal Study found that depression was linked to reduced voting—especially among those who were less wealthy^16^. It is uncertain why we did not find this in the current study although the fact that older adults had 2.2 times higher odds for never voting in Model 1 indicates the importance of the variables we adjusted for in the analysis in mediating the psychological distress-never voting association in this age group.

The association between psychological distress and never voting and past voting only was observed only in hybrid political regimes with country-specific analyses indicating that these results were mainly driven by Georgia and Armenia respectively. Both countries followed a similar economic and political course in the 1990s and did not diverge until the 2003 Rose Revolution in Georgia^52^, which subsequently brought the United National Movement (UNM) to power with plans to liberalise the economy while also strengthening state institutions and reducing corruption^53^. However, while the latter was largely successful, the economic reforms impacted many people negatively. The unemployment rate rose from 11.5 to 16.3% in the period from 2003 to 2010^54^, almost one-quarter of the population (24.7%) were living in poverty in 2009^55^, inequality also rose sharply while 30% of the population were undernourished in the period from 2007 to 2009^53^. Against this backdrop it is possible that economic hardship (poverty/unemployment) might help explain the association we observed between psychological distress and never voting in Georgia. Specifically, an earlier study identified socioeconomic issues (jobs and poverty) as the primary concerns of Georgian voters, with individuals who assessed the government’s policies negatively being more likely not to vote^56^. Given that other research has also shown that individuals who are unemployed^57^ or who are poor^58^ are less likely to vote, it can be speculated that at least some element of Georgia’s non-voters are people who fall into these categories. If that is the case, it might help explain the link with poorer mental health as both unemployment and poverty have been linked to an increased risk of psychological distress^59^, although it should be mentioned that our analyses were adjusted for household financial situation. It is possible that similar factors might also underlie the association with past voting only in Armenia, as in 2010 almost one in five adults were unemployed^60^ while 35.8% of the population were living in poverty^61^. Indeed, a later survey among Armenian residents in 2014 revealed that 83.4% of them thought the country was moving in the ‘wrong’ direction, with 71.9% stating that the low level of economic development and poverty were the priority issues that needed addressing^62^.

This study has several limitations. We lacked information on voting behaviour in different types of election (e.g. local and national) and it is possible that the participants were referring to different types of election when responding. This might be important as an earlier study from Canada found that mental health affected voting more at the local level than the national level^18^. Future research should therefore collect information on the most recent elections at all levels to better determine the association between mental health and voting behaviour and intentions in these countries. We also cannot discount the possibility that some respondents may have reported their voting behaviour incorrectly as voting is regarded as a sensitive issue and some previous studies have found that voting can be overreported^63^. Similarly, the survey used questions that combined reports of actual voting behaviour with future voting intentions. This may have been problematic as many people who report that they will vote in the future do not actually vote^64^. Indeed, as there is some indication that a future intention to abstain from voting may be a better predictor of future voting behaviour^64^, we focused our analysis on those who reported that they would not vote in the future. Nonetheless, it is possible that respondents might have either deliberately or unintentionally misreported their previous voting behaviour and future voting intentions and this might have biased the observed associations. Having data on actual voting behaviour, validated with official records where possible, would be an ideal for future studies. It is also possible that other potentially important factors were not included in the analysis. For example, the patriarchal values common in these countries^65,66^, might underpin both women’s non-voting and their poorer mental health. In particular, in 2010 from a list of 134 countries our study countries (excluding Belarus) ranked from sixty-fifth (Kyrgyzstan) to one hundred and nineteenth position (Georgia) in terms of women’s political empowerment^67^. It is possible that seeing comparatively few women in positions of political power might disincentivise some women from voting, and that the wider societal (and home-based) inequality that underpins this might also affect women’s mental health. Thus, future studies should collect information on a wider range of societal and cultural factors. It is also important to note that the scale we used to measure psychological distress has not been previously validated. Although it contained items that are similar to those seen in other, commonly used measures of psychological distress^68,69^, it is possible that our results might have differed had other measures been used. It will be important for future research to examine the association between mental health and voting using measures that have been validated in all of our study countries. In addition, in the current study the analysis was only adjusted for factors occurring in adulthood (as no information was collected on childhood characteristics) even though one recent study found that childhood conduct problems were also associated with decreased voting in adulthood even after controlling for adult psychiatric morbidity^70^. This suggests that it may be necessary for future studies to focus on both internalising and externalising problems when examining voting behaviour, and also, that the use of a life course perspective would be of value. It should also be noted that this study examined the association between individual characteristics and voting. However, governments have many ways to influence voting including forms of voter suppression such as measures that disproportionately disenfranchise certain groups for example, by closing or relocating polling stations so as to make it difficult to vote^71^, through to direct voter intimidation^72^. As such methods have also been documented in our study countries^73–75^, where not voting may even have potentially detrimental consequences^73^, we cannot rule out the possibility that this might have acted to obscure the association between mental health and voting behaviour. Finally, as this study was cross-sectional we were not able to establish causality nor were we able to determine the directionality of the observed associations. As the presence of depression might affect the reporting of prior voting behaviour and future voting intentions, it is possible that the associations we observed are potentially spurious. Longitudinal research is thus needed to further clarify the association between mental health and voting behaviour.

## Conclusion

In conclusion, this study used data from nine FSU countries that were collected in 2010/11 and found that psychological distress was associated with not voting in elections in the pooled sample, that this association is especially strong in women and working-age adults and is seen in hybrid but not in other types of political regime. As poor mental health disproportionally affects those with a lower socioeconomic status, non-participation in elections may result in increased political inequality^13^, further disenfranchising those who are most socially and economically vulnerable. This can create a cycle of decline, with deteriorating health feeding into a sense of disengagement and hopelessness^76^, which in turn may further undermine health. It is also worth noting that since the time of our survey some of these countries have moved further away from democracy. Specifically, according to the 2022 Democracy Index, Ukraine now ranks as a hybrid regime, while Russia and Kyrgyzstan are categorised as authoritarian regimes^77^. Future research will thus be necessary in our study countries to determine whether the results obtained in this study are replicable or merely represent a specific point in time given the changing socio-political circumstances seen in many of these countries.

## Methods

### Study participants

Data came from the Health in Times of Transition (HITT) survey. This cross-sectional survey was undertaken in Armenia, Azerbaijan, Belarus, Georgia, Kazakhstan, Kyrgyzstan, Moldova, Russia, and Ukraine in 2010/2011. In each country a nationally representative sample was obtained by using multi-stage random sampling with stratification by region and settlement type (urban/rural). Random route procedures were used to select individual households from within the primary sampling units (PSUs) (approximately 100–200 per country). Within each selected household one individual aged 18 or above was randomly chosen to participate in the survey (determined by the nearest birthday). Trained interviewers undertook face-to-face interviews in the participants’ homes to obtain information using a standard questionnaire with items presented in each country’s language. Respondents could answer in either their own national language or Russian in every country with the exception of Russia and Belarus, where only Russian was used. Exclusion criteria included being hospitalised, institutionalised, incarcerated, homeless, in the military, or intoxicated at the time the survey was conducted. Across the nine countries a total of 18,000 respondents were included in the study. In six countries the sample size was 1800 respondents while in three countries the sample size was larger. Specifically, in Russia and Ukraine the sample sizes were 3000 and 2000 persons respectively, in order to take into account their larger and more regionally diverse populations. In addition, in Georgia the sample size was 2200 following a 400 person booster survey that was undertaken towards the end of 2010 in order to make the sample more representative. Across the nine countries the survey response rates ranged from 47% (Kazakhstan) to 83% (Georgia)^78^.

### Ethical permission

Permission for the study was provided by the ethics committee at the London School of Hygiene and Tropical Medicine. Ethical permission was also obtained from the Open Health Institute (for Russia); the GORBI Foundation (for Georgia and also for Armenia and Azerbaijan); the Centre for Social and Political Research, Belarusian State University (Belarus); the East-Ukrainian Foundation for Social Research (Ukraine); the Independent Sociological and Information Service “Opinia” (Moldova); and the Centre for Study of Public Opinion (CIOM) (Kazakhstan also responsible for Kyrgyzstan). The study was undertaken in accordance with the 1964 Helsinki Declaration and its subsequent revisions. Participants were provided with a written and verbal explanation of the study, including their right not to participate or to withdraw without giving a reason, and that doing so would have no effect, positive or negative, on them or their household. Written informed consent was obtained from all participants.

### Measures

#### Psychological distress

This was assessed with a 12-item scale that was first used in the Living Conditions, Lifestyles and Health (LLH) survey that was undertaken in eight FSU countries in 2001^48^. Respondents were asked if “In the recent several weeks have you experienced the following problems?”: (1) Been unable to concentrate on whatever you are doing; (2) Insomnia; (3) Felt constantly under internal strain; (4) Felt you couldn't overcome your difficulties; (5) Losing confidence in yourself; (6) Nervous shaking or trembling; (7) Frightening thoughts coming in your mind (8) Get spells of exhaustion or fatigue; (9) Feeling stress (10) Impossibility to influence things; (11) Feeling lonely (12) Life is too complicated. In response to each item participants answered either ‘yes’ (scored 1) or ‘no’ (0). Scores could range from 0 to 12 with higher scores indicating increased psychological distress. In order to focus on those with the most severe symptoms and in line with recent research, which has indicated that the prevalence of depressive and anxiety disorders is between 3 and 6% in these countries^79^, in this study we classified individuals in the top 5% of scores (a score of 10 and above) as experiencing psychological distress. This measure has been used previously in these countries and is associated with other measures of psychological ill health^80^. Cronbach’s alpha for the scale was 0.81.

#### Voting behaviour

Respondents were presented with the statement, “There are different forms of political activities. What would you say about your participation in them?” One of the listed forms of political activity was ‘voting’. In relation to this, respondents were presented with five response options and asked to choose one: (i) Did it and will do it in future (i.e., ‘always voting’); (ii) Did it but will not do it in future (i.e., ‘past voting only’); (iii) I did not participate, but I will participate in future (i.e., ‘future voting only’); (iv) I did not participate and I will not participate in the future (i.e., ‘never voting’); (v) Don’t know. As our focus in this study is on non-participation, in the analyses the response option (i) always voting was used as the comparison category, while options (ii) past voting only and (iv) never voting were used separately as outcomes. We decided not to use option (iii) as an outcome in the analysis as previous research has shown that there can be a large disparity between individuals’ expressed intention to vote in the future and their actual future voting behaviour^64^, and it is feasible that this might be even greater among those who have never previously voted. In contrast, few of those who state that they will not vote in future actually vote i.e. intention to abstain may be a more reliable measure of future voting behaviour^64^.

#### Covariates

Information was collected on the sex of the respondents (men, women), while age was a continuous variable that was subsequently divided into three categories: 18–34, 35–59 and ≥ 60 representing young, middle-aged and older adults, respectively. In line with previous studies^81,82^ education was also assessed using three categories, low (incomplete secondary education or below), mid (completed secondary/secondary special education), and high (completed/non-finished higher education). In terms of marital status respondents were categorised as being married/cohabiting, never married, or divorced/widowed. For location, respondents were classified as living in one of five types of settlement: (i) capital of the country; (ii) regional capital; (iii) city (but not country or regional capital); (iv) settlement of an urban type; (v) village^83^. Using this information respondents’ residential location was then subsequently classified as being either urban (settlement types (i)–(iv)) or rural (v). The financial status of each respondent’s household was assessed with a question that asked, “How would you describe the economic situation of your household at the present time?” There were five response options, very good, good, average, bad and very bad, which were subsequently combined into three categories, good/very good, average, bad/very bad. The household composition of the respondents was determined by asking, “Including you, how many people constantly live in this household (including children and adults)?” All those respondents who answered ‘one’ were then categorised as living alone. Respondents were asked to rate their own health using one of five response options, which were then combined into three categories, good/very good, fair, or bad/very bad. Social support was assessed using five questions including, “Is there anyone who you can really count on to listen to you when you need to talk?”, and “Is there anyone who can comfort you when you are upset?” The answers were combined to create a total scale score running from 0 to 5 with higher scores indicating greater social support. We then dichotomised the total score so that individuals with a score of 0 to 3 were categorised as having low social support. This scale, with slightly different answer options, has been previously used to assess social support in the British Household Panel Survey^84,85^. Cronbach’s alpha for the scale was 0.85. Finally, as previous research has indicated that political trust/distrust is linked to both voting behaviour^86–88^ and mental health^89,90^ we also included a measure of political distrust in the analysis. This was assessed with a question asking “Please tell me on a score of 1–10, where 10 means complete trust and 1—absolute distrust, how much you personally trust each of the following institutions:” (i) president of the country; (ii) government; (iii) parliament; (iv) county/regional council; (v) mayoralty; (vi) political parties. After reverse-coding the scores, we calculated the mean of the 6 items ranging from 1 to 10 with higher values indicating greater institutional distrust. Cronbach’s alpha for the scale was 0.93.

### Statistical analysis

To account for study variables with missing information, we first used multiple imputation to generate 20 datasets. Specifically, we used the chained equation method and used linear, logistic, ordered logistic and multinomial logistic regression models for continuous, binary, discrete or categorical variables, respectively. We used Rubin’s rules and combined imputation estimates. Descriptive statistics were then calculated for the total sample, stratified by the respondents’ voting behaviour status. Next, logistic regression was used to examine the relationship between psychological distress and two forms of voting behaviour in the pooled sample. Five models were used to examine the associations. Model 1 examined the bivariate association between psychological distress and voting. In Model 2 the sociodemographic variables sex, age, education, marital status, household financial situation, living alone, and location were included in the analysis. Model 3 included the same variables as in Model 2 with the addition of the self-rated health variable. Model 4 included the same variables as in Model 3, and also low social support. The fully adjusted Model 5 included the same variables as in Model 4 plus political distrust. Sex- and age-specific analyses were then performed using the same analytic model building process described above. Finally, we conducted analyses using the same model building process to examine the association between psychological distress and voting behaviour in different types of political regime and in each of our study countries.

SPSS version 24 and STATA version 17 were used to conduct the analyses. In the pooled analyses all models have country dummy variables^91^. The results are presented as odds ratios (OR) with 95% confidence intervals (CI). The level of statistical significance was p < 0.05 (two-tailed).

### Supplementary Information

Supplementary Information.

## Data Availability

The data will be provided by the first author (email: amstick66@gmail.com) on request for 5 years post-publication.
