# Supplementary material for: Psychological distress and voting behaviour in nine countries of the former Soviet Union
Source: Sci Rep. 2023 Dec 19;13:22709. doi: 10.1038/s41598-023-49071-8 (PMC10733372; doi:10.1038/s41598-023-49071-8)
Supplement: Supplementary file 1 — Supplementary Information. [file 41598_2023_49071_MOESM1_ESM.pdf]

Supplementary information

Psychological distress and voting behaviour in nine countries of the former Soviet Union

Andrew Stickley, Tomiki Sumiyoshi, Naoki Kondo, Mall Leinsalu, Yosuke Inoue, Vladislav Ruchkin, Jae Il Shin, Martin McKee

Appendix 1 Economist Intelligence Unit's Index of Democracy

The types of regimes found in the countries of the former Soviet Union are described below using the description provided by the Economist Intelligence Unit to delineate these differing regimes.*

Flawed democracies: Countries have free and fair elections and although there can be problems (e.g. infringements on media freedom), basic civil liberties are respected. However, other aspects of democracy have major weaknesses, including problems in governance, a political culture that is underdeveloped and low levels of political participation.

Hybrid regimes: Countries have substantial irregularities in elections that often prevent them from being both free and fair. Opposition parties and candidates may commonly experience government pressure. The serious weaknesses observed in political culture, government functioning and political participation are more prevalent than in flawed democracies. The rule of law is weak and corruption tends to be extensive. Civil society is weak, the judiciary is not independent and the harassment of and pressure on journalists is typical.

Authoritarian regimes: Political pluralism is absent or heavily restricted in these states. Many countries in this category are out-and-out dictatorships. If formal institutions of democracy exist, they will have little substance. If they do occur, elections are not free and fair. Abuses and infringements of civil liberties are disregarded. Media are usually state-owned or controlled by groups connected to the ruling regime. Criticism of the government is repressed and censorship is pervasive. The judiciary is not independent.

*From: Economist Intelligence Unit., 2010. Democracy Index 2010: Democracy in Retreat – a Report from the Economist Intelligence Unit. London, EIU, pp. 31-32.
